# Identification of Phase Transformations in Alloy and Non-Alloy Steel During Austempering Using Acoustic Emission and Neural Network

**DOI:** 10.3390/ma18102198

**Published:** 2025-05-10

**Authors:** Małgorzata Łazarska, Zbigniew Ranachowski, Janusz Musiał, Tomasz Tański, Qingshan Jiang

**Affiliations:** 1 Faculty of Materials Engineering, Kazimierz Wielki University, Chodkiewicza 30, 85-064 Bydgoszcz, Poland; 2 Institute of Fundamental Technological Research, Polish Academy of Sciences, Pawińskiego 5B, 02-103 Warszawa, Poland; zranach@ippt.pan.pl; 3 Faculty of Mechatronics, Kazimierz Wielki University, Mikołaja Kopernika 1, 85-074 Bydgoszcz, Poland; janusz.musial@ukw.edu.pl; 4 Faculty Institute of Engineering Materials and Biomaterials, Silesian University of Technology, Konarskiego 18A, 44-100 Gliwice, Poland; 5 College of Marine Equipment and Mechanical Engineering, Jimei University, Xiamen 361000, China; 201961000131@jmu.edu.cn

**Keywords:** bainite, martensite, austempering, acoustic emission (AE), neural networks

## Abstract

This research was carried out for selected alloy (bearing) and non-alloy (tool) steel. The steels were subjected to austempering. The hardening temperature range was from 100 °C to 180 °C. The use of acoustic emission in connection with the artificial neural network (ANN) enabled the analysis and identification of phase changes occurring in steels during austempering. Classification of acoustic emission events was carried out with the help of their energy values and with the use of an artificial neural network. On this basis, it was observed that in the process of isothermal hardening of steel at the applied temperatures, complex transformations of austenite into martensite and bainite occur. In addition, it was found that the research methods used enabled the identification of signal components originating from the phase transformation causing the formation of thin-plate martensite midrib. The use of acoustic methods in the field of bainitic transformation creates the possibility of their application in the industry.

## 1. Introduction

The accelerated pace of technological advancement drives the pursuit of innovative, more effective, and safer technologies, also in relation to steel products. The proper selection of heat treatment methods has a fundamental impact on the optimal use of steel structural components. The current market places pressure on accurate analysis and control of the condition of materials that are the subject of the design and manufacturing processes of devices. To produce products with the desired functional characteristics, it is essential to understand the phenomena that occur during heat treatment. As a result, researchers are seeking methods to track the kinetics of phase transformations in steels during the austempering process.

Nowadays, various methods of non-destructive testing of materials are known. Among them, the acoustic emission method occupies a very important place. The use of this research technique makes it possible to track not only cracking and damage processes, but also to study phenomena occurring during the heat treatment of steel. A current AE apparatus, equipped with multiple channels for simultaneous signal recording and modules for analysing this signal in the time and frequency domains, creates the possibility of conducting effective measurements. Acoustic emission tests were conducted in many laboratories [1,2,3,4,5,6,7,8,9,10]. The phenomenon of acoustic emission in metals and their alloys depends on a number of different factors, often including very intricate and complicated physical phenomena. A lot of theoretical and experimental work has been carried out to clarify the mechanism of the occurrence of AE in materials. The processes studied are related to phenomena of different scales: sub-, micro-, and macroscopic. Since EA is most readily observed in solids and, in addition, in more brittle than ductile bodies, they are therefore best studied. The main sources of acoustic emission in metals and their alloys are considered to be the movement of dislocations as a result of plastic deformation, the formation of twins, and displacement-type phase transformations. In a number of experiments, deformation of the crystal microstructure of steels in austenite–martensite and austenite–bainite type transformations has been found to be the source of AE signal generation [11].

Artificial neural networks are used in solving complex algorithmic problems and analysing large data sets. They are characterised by high computational efficiency, which reduces the time consumption of calculations and optimises the use of physical resources of computing systems. Artificial neural networks are successfully used in information processing, speech recognition, spatial object classification, and medical diagnostics. They are also used to analyse various processes and changes in the physical properties of technical objects. Neural networks have also been used in research on the properties of materials, including as a tool for analysing the AE signal generated by a large number of sources that evolve as a function of time [2,5].

A prerequisite for producing materials with suitable performance properties is a sound knowledge of the physical processes occurring during the production process. A systematic study of the phenomena occurring during the decomposition of overcooled austenite for eutectoid steel was initiated by E. S. Davenport and E. C. Bain in 1930 [12]. These researchers proposed the use of isothermal transformation as a technique to study phase transformations as a function of time and temperature. Davenport and Bain presented the phase transformations taking place depending on temperature and time during the isothermal transformation in the time–temperature–transformation (TTT) diagram. This diagram shows the occurrence of characteristic C-type curves separating the areas of occurrence of individual austenite decomposition products. The TTT diagram became the basis for many further studies of the isothermal process. As a result, the development of dilatometric equipment followed and a number of theoretical research works related to the mechanism of growth and development of the emerging phases were created. Currently, TTT diagrams are commonly used in the heat treatment processes of steels. The bainitic transformation is still the least studied of all austenite transformations in steels, so interest in it remains considerable among researchers. Significant achievements in the study of the phenomenon of accelerated onset of transformation were obtained by H. Okamoto and M. Oka [13,14], performing dilatometric studies for 0.85–1.8% C steels. Based on the results obtained, it was observed that the “swing back” effect of deformation of the curves separating the areas of formation of different phases increased with increasing carbon content in the steel. It is presumed that this phenomenon is influenced by the formation of isothermal thin-plate martensite. This product of austenite decomposition is characterised by the occurrence of large carbides with a characteristic shape inside bainite plates. A significant contribution to the understanding of the phenomena associated with phase transformations was made by H.K.D.H. Bhadeshia [15,16,17], who developed a model of the mechanism of bainitic transformation. In this model, the cause of bainitic ferrite formation is the mechanism of cooperative shearing of iron atoms due to changes in the volume of austenite transformation products. After heat treatment, steel may have a mixed microstructure consisting of various transformation products.

The use of the acoustic emission method in combination with an artificial neural network made it possible to analyse and identify the phase transformations occurring in steels during austempering. The experiments conducted showed that the acoustic image is the result of structural changes occurring during the austempering of steel. Acoustic emission (AE) is a research method, similar to conventional techniques such as dilatometry, used for monitoring phase transformations. However, AE differs from conventional methods in terms of sensitivity, application, and the type of data obtained. Acoustic emission detects acoustic waves emitted during microstructural changes in a material, such as cracking, twinning reorientation, or phase transformations. Unlike dilatometry, it is more sensitive to rapid and localised changes in structure. It can detect even very subtle transformations that do not cause significant volumetric changes. Furthermore, it enables the monitoring of dynamic processes, such as martensitic transformations in steels. For dynamic transformations associated with a distinct release of energy, acoustic emission can be more accurate and sensitive than conventional testing methods. Additionally, a new research approach in this study was the use of a simpler method than the neural network for event classification, using the AE event energy values. The combination of the applied research methods opens up new possibilities, especially in cases where signals are complex and difficult to interpret using classical techniques. Artificial neural networks (ANNs) handle large amounts of data and can be used to classify results. AE signals are often complex and nonlinear, and ANNs are well-suited for analysing such data. Moreover, neural networks can detect patterns that might be overlooked in traditional signal analysis. Monitoring phase transformations in alloy and non-alloy steels using these methods enables the identification of the start and end points of martensitic and bainitic transformations. There is a growing effort to implement and apply these technologies in industrial settings. The benefits for industry include more precise quality control through direct identification of phase transformations. Additionally, the system could provide early warnings and immediate detection of anomalies (e.g., incomplete martensitic transformation), helping to prevent defective batches. Thanks to rapid error detection, the process can be stopped or corrected before producing large quantities of faulty products. Data analysis using ANN also improves understanding of transformation processes and allows for optimisation of parameters (e.g., time and temperature), leading to energy and material savings.

## 2. Research Methodology

Phase transformations occurring during the decomposition of overcooled austenite in alloy and non-alloy steel were studied. The chemical composition of the steels is shown in Table 1 and Table 2. In order to identify the transformations after austempering, the parameters of the AE signal subjected to filtering were analysed using a neural network.

In the first stage of the research, a heat treatment operation was carried out, consisting of austempering of samples with a diameter of 45 mm and a thickness of 2 mm of selected steel grades. The steel samples were subjected to heat treatment in a range of different temperatures, approximately below and above *M*_s_, suitable for the material grade. The tests were carried out in a specially prepared test stand, equipped with an apparatus for EA measurements. The recorded acoustic emission signals were then processed and analysed using an artificial neural network. In the last stage, the microstructure of properly prepared steel samples after austempering was reviewed.

**Figure 1:**
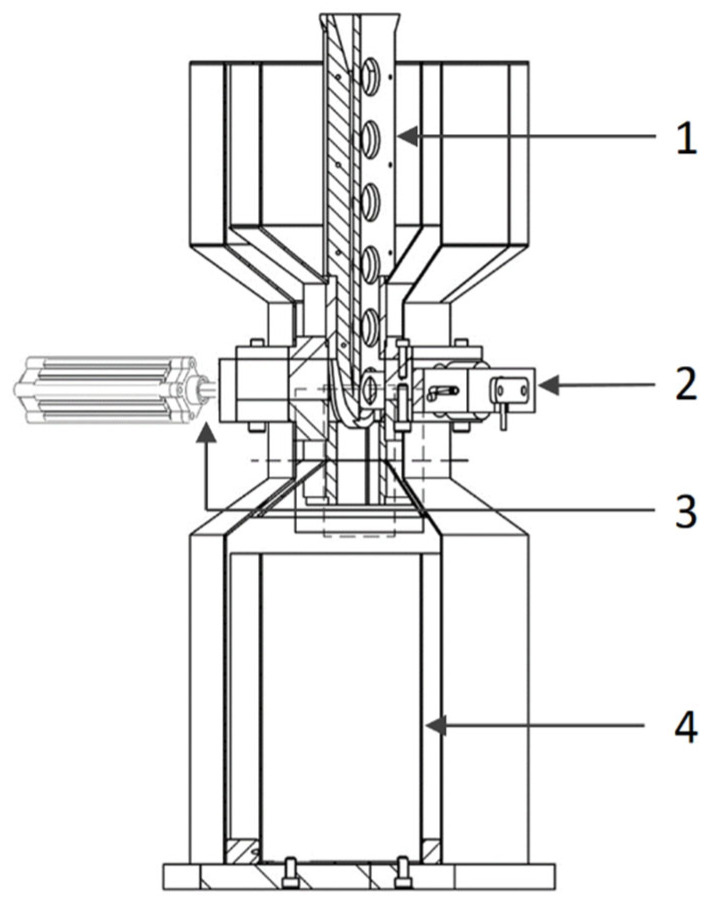
Schematic of the test stand for austempering. Austempering in oil with simultaneous recording of acoustic effects. 1—guide, 2—ultrasonic sensor, placed on the left of the pair of waveguides, 3—right of the pair of waveguides and a fragment of the pressing mechanism, 4—oil tank.

The samples were austenitised in a chamber furnace at a temperature of 950 °C for 1800 s. The austenitisation parameters were designed in such a way that it was possible to dissolve carbides in a significant amount and reduce the temperature *M*_s_. The given conditions enabled the growth of retained austenite during steel hardening and the reduction of deformations of the finished elements. The recording of the acoustic signal during austempering was carried out on a specially prepared test stand, shown in Figure 1.

The main element of the stand is a thermostated tank filled with oil. A guide is installed inside the tank to move hot samples. The temperature is measured using two thermocouples immersed in oil, integrated with the control system (temperature controller) and a band heater. At the end of one of the pair of waveguide pins, an ultrasonic sensor is attached to record the acoustic emission signal generated during the austempering process. A pneumatic actuator is attached to the second pin, enabling the correct pressure of the sample against the waveguides. Thanks to the use of a material with appropriate thermal resistance, the temperature of the outer ends of the waveguides does not exceed 100 °C during the austempering process, which is within the safe operation range of the AE sensor. The waveguides are made of 4H13 steel, for which tests were performed to exclude the occurrence of phase changes in the temperature range in which these waveguides are operated. The quenching oil is heated using a band heater installed in the lower part of the tank. The station control system includes a relay controlling the pneumatic actuator, air pressure indicators on the actuator and reducer, and two oil temperature regulators in the tank.

The study was performed in the following stages:

- Austenitisation of the sample in a chamber furnace at 950 °C for 1800 s.
- Placing the sample in a test stand (in a special guide), filled with oil heated to an appropriate temperature (100–180 °C).
- Pressing with pins that serve as waveguides to record the AE signal.
- Registration and recording of the AE signal within 4 min.

**Figure 2:**
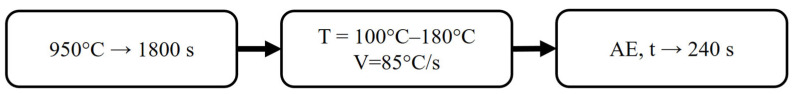
Diagram of the heat treatment process, with the marked time interval (t) during which the acoustic emission signal was recorded.

A diagram of the heat treatment process at the austempering station, with the time interval t marked, in which the acoustic emission signal was recorded, is shown in Figure 2.

**Figure 3:**
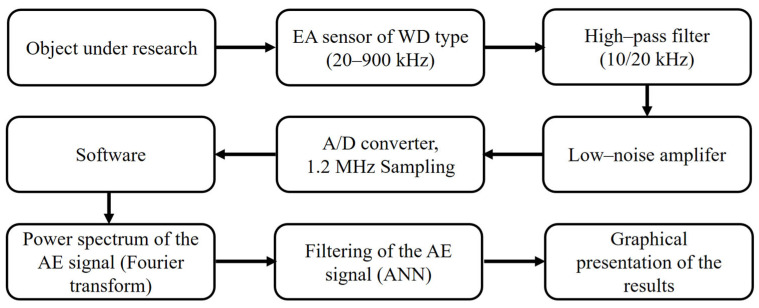
Diagram of the AE test stand.

The AE signal generated as a result of phase transformations occurring during austempering of steel was recorded using the original measurement set. The diagram of the measuring device is shown in Figure 3.

The AE signal was recorded using a differential, wideband WD type sensor (20–900 kHz). The sensor was connected to a low-noise amplifier whose operating band noise level did not exceed 50 microvolts of peak-to-peak voltage. The amplifier allowed the signal to be amplified in the range of 20–60 dB. During the measurements, the maximum possible signal amplification was used. A high-pass filter connected to the amplifier’s output made it possible to eliminate background noise coming from the equipment’s surroundings. The amplified and interference-free AE signal was transmitted to a PC with a built-in 12-bit analogue-to-digital converter card. An ADLINK type 9812 transducer (ADLINK Technology Inc. Taoyuan City, Taiwan) with a sampling frequency of 1.2 MHz was used. The presented measurement system enabled the recording of the AE signal for 240 s. After completing the registration, a file in the wav format was generated on the hard disk of the control computer, the size of which was 576 Megabytes.

The measurement system was equipped with software to analyse the recorded signal. The software kit included the following:

- WIDMO program (The software was developed at the IPPT PAN in Warsaw, Poland) for graphical presentation of the AE signal in the form of a spectrogram, i.e., in the time–frequency system.
- a set of programs for filtering the recorded signal using a neural network and, subsequently, for graphical presentation of the results of this operation.

The STFT (Short Time Fourier Transform) algorithm with a Hamming window was used to generate the spectrogram in the WIDMO program. The program enabled visualisation of the AE signal with a time domain resolution of 15 ms. Each 15 ms signal segment contained 18,000 signal samples. A total of 1647 samples were used to construct the spectrogram, in which the maximum energy of the AE signal was detected.

Software consisting of three programs (developed specifically for this research) was used for signal filtering. The first program divided the signal into segments lasting 7.5 ms and determined their energy. Spectral characteristics were created, which served as feature patterns input into the neural network. The second program carried out the neural network training procedure and monitored the network’s status during training. The third program was used to perform the classification of recorded AE events. This program allowed the loading of the neural network’s weight coefficients after the training procedure and the classification of feature vectors (*c_n_*) provided at the network’s input.

For the samples studied in this work, the ranges of recorded energy levels of AE events were as follows:

- High-energy events—above 10,000 pJ
- Medium-energy events—in the range of 1000 to 10,000 pJ
- Low-energy events—below 1000 pJ

The analysis of the recorded AE signal consisted of extracting the components, that is, the AE events, from this signal. The prepared software made it possible to identify AE events with certain values of energy and frequency characteristics within the population of events recorded during each of the austempering processes carried out. The energy of events [J] was determined according to the following formula:

(1) $$E_{AE}=\frac{1}{2Z}A_{i}^{2}\;∆t$$

where *A_i_*—voltage amplitude during the generation of the *i*-th event [V], *Z*—electrical impedance of the EA sensor (*1000 [V/A]*), and Δ*t*—duration of the *i*-th event [s].

It was assumed that during a specific phase transformation, AE events with similar parameters are generated from the transformations occurring during the austempering process. Determining the frequency response involved determining a set of parameters called spectral characteristics based on signal samples included in the AE event. In the case of an analogue signal *v*(*t*) in a selected time interval, it is possible to transform it into a function depending on the frequency parameter. Assuming the absolute integrability of the function *v*(*t*), it can be represented as an integral of the spectral density function *A*(*ω*) according to the Fourier transform.

(2) $$\upsilon \left(t\right)=\frac{1}{\pi }\int_{0}^{\infty }A\left(\omega \right)\sin\left[\omega t+\phi \left(\omega \right)\right]d\omega$$

where *φ* is a parameter representing the phase of the transformed signal.

For a discrete signal, its samples were divided into segments aligned with time intervals suitable for analysis (each containing 18,000 samples). Within these segments, the discrete spectral density function was evaluated. The algorithm that converts the signal samples into spectral density coefficients *c_n_*, i.e., *v_EA_*(*mT*_1_) ⇒ *c_n_* (*ω*), was applied using the following approximate formula:

(3) $$c_{n}\approx \frac{1}{N}\sum_{m=0}^{N-1}v\left(m\cdot T_{1}\right)\cdot \mathrm{mod}(e^{-\frac{jn2\pi m}{N}})$$

where *j* stands for √(−1) and mod stands for the absolute value of the expression in brackets.

The analysis of the recorded AE signal using a neural network was carried out in three stages, using three programs. In the first one, the recorded signal was segmented into subsets with a duration of 7.5 ms. Then, for each subset, the forty-element spectral characteristic of the dominant AE event, i.e., the event with the highest energy, was determined. The first program made it possible to determine the energy of AE events recorded during the austempering process. A detailed analysis of these data allowed obtaining information that at the first stage of the process, i.e., for the first 20% of its duration, events with a high energy level dominate in the population of registered AE events. During the next 40% of the duration, medium-energy events dominate, followed by low-energy events close to the acoustic background energy. This program also made it possible to determine the spectral characteristics of events specified by the operator. In the second stage of AE signal processing, four pairs of patterns required to carry out the network training process were determined for both types of tested steel. These were the spectral characteristics of the following events. The spectral characteristics of these patterns, i.e., forty-element vectors with *c_n_* coefficients, were then processed into a form enabling their introduction to the input of the neural network. The neural network used had 4000 binary inputs (i.e., zero meant no signal, and one meant the appearance of a signal). Therefore, a logarithmic function was applied to the *c_n_* coefficients so that the resulting numbers would lie within the 0 to 10 range. Forty *c_n_* coefficients, scaled in the range of 0–10, were introduced to the network inputs. The structure of the neural network was composed of neurons distributed across two layers. The first hidden layer contained an array of 200,000 weight coefficients mapping the connections between the units of the input layer and 50 units of the next layer. The second hidden layer contained an array of 3000 weight coefficients mapping the connections between the units of the previous layer and the six units of the output layer. At the network’s outputs, a signal was produced based on the similarity between the input vector and the stored patterns.

The spectral characteristics of the patterns, i.e., forty-element vectors with coefficients *c_n_*, were processed into a form suitable for input into the neural network.

The process of memorising (one or several) patterns was carried out automatically using an iterative algorithm. Considering the feature vectors processed in the network as sequences of zeros and ones, the relationship between the input signal *y_j_* of a single neuron and the output signal *y_i_* can be written as follows:

(4) $$y_{i}^{\left(t+1\right)}=\theta +(\sum_{j}w_{ij}+y_{j}^{\left(t\right)}-\mu_{i})$$

where *θ*—activation function, most often in the form of a sigmoid 1/(1 + *exp*(−*x*)), *w_ij_*—weight of the connection between the *i*-th neuron and the *j*-th neuron, and *μ_i_*—threshold activation level of the *i*-th neuron.

The network training procedure consisted of applying a series of repetitions of the algorithm described by Formula (4) sequentially for all feature pattern vectors of the signal, with the training sequence being varied, as during the training of a new pattern, previously learned patterns are partially “forgotten”. During this procedure, an association is formed between the maximum output signal of one of the six neurons in the last layer of the network and one of the types of AE signals. The goal of the training process was to achieve such a distribution of neural connection weights that, upon presenting a set of spectral coefficient vectors of the power density of the signal from a segment of the recorded AE signal, the neuron that had memorised the most similar feature vector would respond with the maximum output signal. In practical network training, it is necessary to use three pairs of patterns (patterns of high-power AE signals; patterns of medium-power AE signals in A1, B1, and C1; and patterns of low-power AE signals in A2, B2, and C2, including background noise in A3, B3, and C3).

The artificial neural network was trained using the back propagation algorithm [18].

(5) $$∆w_{ij}^{\left(k\right)}=\eta_{1}+(\frac{d\theta \left(E_{i}\right)}{dE})x_{j}\delta_{i}^{\left(k\right)}+\eta_{2}m_{ij}^{\left(k+1\right)}$$

The network training sequence consisted of repeatedly applying the procedure presented by Equation (5) to individual patterns in alternating cycles.

The second program was used to carry out the procedure of training neural networks, monitoring the network’s state during the learning phase and evaluating the network outputs after spectral patterns of AE events were fed into its input.

During the network training procedure, an experimentally selected sequence of patterns was fed to its inputs, and the network outputs were adjusted so that one of them reached the highest signal level whenever a specific pattern appeared at the input. In this setup, the signal present at the network outputs served to classify the vectors introduced at the input. The relation between the input vector *X* and the output vector *Y* for each layer of the network can be illustratively represented as follows:

(6) $$Y=W^{*}X$$

where *W* is a matrix containing the weight factor, and * denotes the operation of mapping the network layer’s inputs to its outputs.

Upon the conclusion of the learning process, the neural network became capable of classifying sets of spectral characteristics *c_n_* provided at its input, based on how similar they are to the previously learned patterns: A, B, and C. The last program was used to carry out the classification procedure of the registered AE events. This program had the ability to load the coefficient weights of the neural network after the learning procedure and classification of the vectors *c_n_*, fed to the network input. The results of this operation were presented in the form of a time dependence of the frequency of events consistent with the selected pattern.

## 3. Results of the Research

### 3.1. Results of Testing the Acoustic Emission Signal Before Filtration

**Figure 4:**
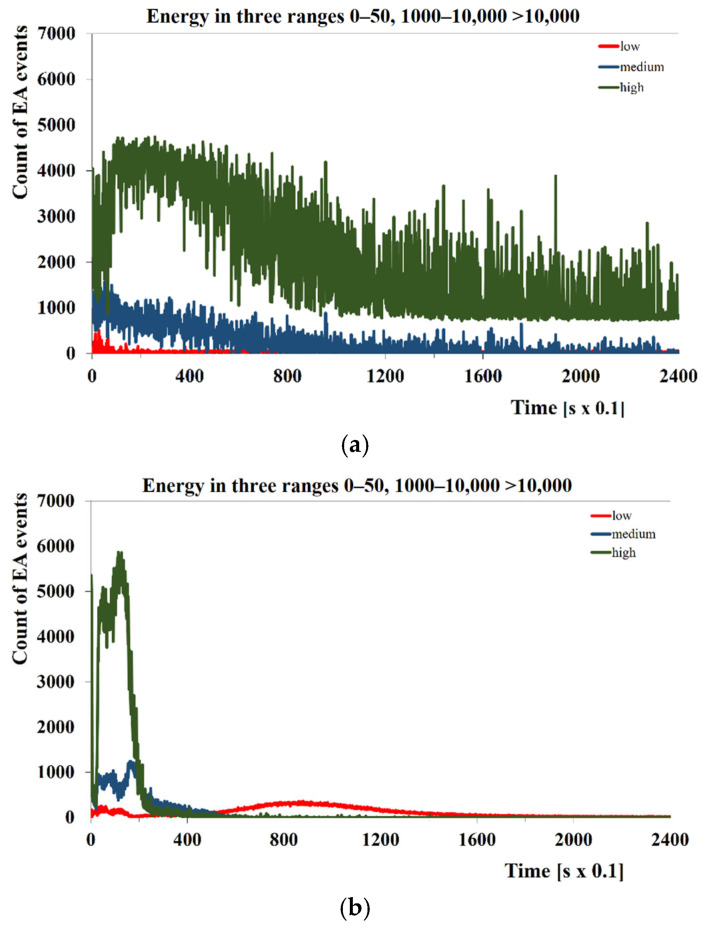
Classification of events using their energy values, for (**a**) 100CrMnSi6-4 steel (160 °C) and (**b**) C105U steel (130 °C).

As a result of the austempering process of alloy and non-alloy steel, AE signal records were obtained for all assumed process temperatures. The tests were carried out in the temperature range of 100–180 °C, below, in the surroundings, and above the temperature *M*_s_ of the tested steels (based on dilatometric tests, it was assumed that the temperature *M*_s_ for bearing steel is 159 °C and for tool steel 132 °C). Based on these relationships, it is possible to determine the duration of the isothermal transformation, but without the possibility of identifying the two phases of bainite formation. Figure 4a,b shows the time dependence of the frequency of occurrence of low-, medium-, and high-energy AE events. The only criterion for classifying the occurrence of the presented events was their energy in the AE signal records. Figure 4 shows that the time of occurrence of the maximum frequency of the generation of high-energy events is earlier than the moment of the maximum of the generation of medium-energy events. The maximum of the generation of low-energy events is significantly shifted in time compared to the corresponding higher-energy maximum.

The method of classifying acoustic emission events using their energy values enabled a preliminary analysis of the received signal and AE events. Based on the obtained time graphs, it can be concluded that at least two phase transformations occur during the hardening process, because these graphs show two maximum frequencies of events with specific energy levels.

**Figure 5:**
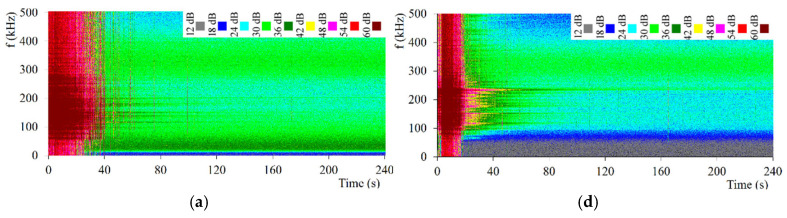

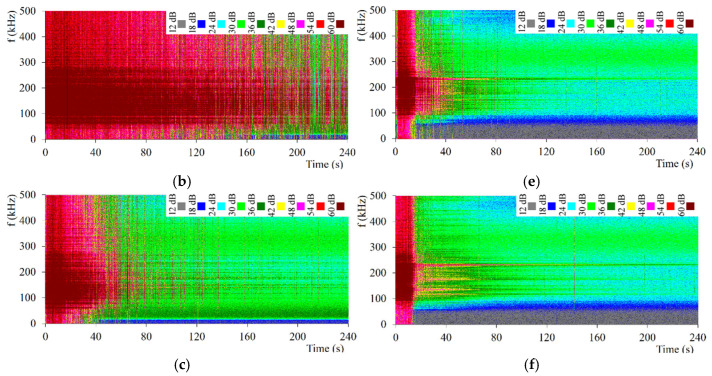
Spectral characteristics determined by the artificial neural network for 100CrMnSi6-4 steel (**a**) at 130 °C, (**b**) at 160 °C, and (**c**) at 180 °C, and for C105U steel (**d**) at 100 °C, (**e**) at 130 °C, and (**f**) at 160 °C.

Then, three pairs of spectral characteristics patterns were determined, selected from the areas of high, medium, and low signal energy, and a pair of acoustic background patterns. The spectral characteristics of three patterns differing in the energy of the AE signal are shown in Figure 5a–f. These figures show significant differences in the presented spectral characteristics: the maxima of these characteristics fall at different frequencies in the case of patterns generated for different energy values of the generated events. The visible maxima cover frequency ranges that differ in the range of these frequencies.

Based on the spectrograms presented in Figure 5a–f, it can be concluded that the maximum energy of the AE signal—taking into account the generation time of the AE signal—occurs during the transformation at a temperature close to *M*_s_. The reason for this effect can be explained by the fact that at a temperature close to *M*_s_, there is an equilibrium state between the enrichment of non-convertible austenite in carbon and the precipitation of ε carbide in the bainite plates, which significantly affects the higher intensity of the processes in which the AE signal is generated. The acoustic emission signal energy for tool steel is lower compared to the signal level and intensity for bearing steel. The presented spectrograms also show that in the case of tool steel, the transformation occurs in a shorter time than in the case of the tested alloy steel. In the case of the tested alloy steel, when the temperature increases from 130 °C to *M*_s_, the signal energy value during the AE transformation increases with the increase in temperature. The strongest acoustic effects occur within 50 s of the process. In the next time interval, single acoustic emission events were recorded. However, after the process duration exceeded 100 s, the signal energy approached the acoustic background level. Further increasing the transformation temperature to 180 °C reduces the energy of the acoustic emission signal. In this case, however, the occurrence of active, low-frequency AE sources in the time interval above 100 s is noteworthy. The decrease in the energy of the AE signal during the transformation in the higher temperature interval can be explained by the decrease in the nucleation rate on the midribs formed.

Moreover, the relationships mentioned above show that, for tool steel, the highest acoustic activity is recorded at a temperature of 130 °C, i.e., at a temperature close to *M*_s_ for this steel. In this range, a large number of events are observed for all adopted energy ranges. However, above *M*_s_, at a temperature of 160 °C, the number of medium energy events is limited, and the events may show a certain degree of similarity, which may mean that only two phenomena occur. However, low energy events for all assumed temperatures are recorded with a similar frequency. This means that the low energies come from the acoustic background. For tool steel, the situation is similar: at a temperature close to *M*_s_ the highest acoustic activity of events is observed. In the process of hardening the bearing steel, high energy AE events were recorded in the frequency range of 100–300 kHz. In the case of tool steel, the frequency range of high-energy events was 100–200 kHz, and the transformation time was significantly shorter.

**Figure 6:**
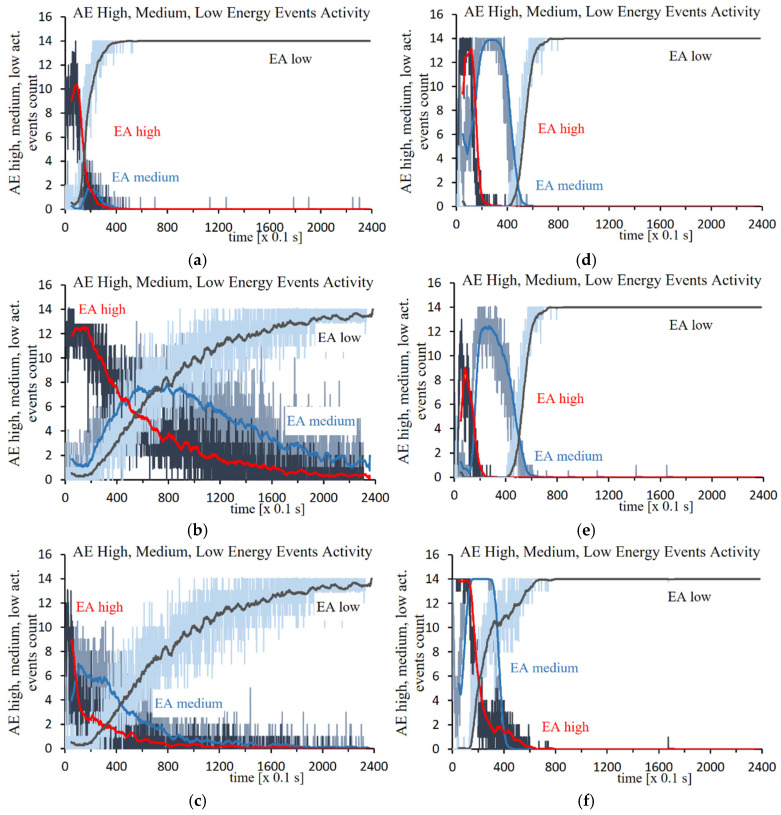
Time dependence of the frequency of AE events correlated with patterns, for high energy AE events, for medium energy AE events, and for low energy AE events, austempering for 100CrMnSi6-4 steel (**a**) at 130 °C, (**b**) at 160 °C, and (**c**) at 180 °C, and for C105U steel (**d**) at 100 °C, (**e**) at 130 °C, and (**f**) at 160 °C.

The use of filtering the recorded signal using the neural network method (Figure 6a–f) made it possible to separate events originating from two different processes.

Signals correlated with previously prepared feature vectors were identified at the outputs of the neural network, which made it possible to monitor phase transformations during austempering. Based on the tests performed for 100CrMnSi6-4 steel, it was found that most events with high acoustic emission energy occur up to approximately 20 s of the signal duration and for temperatures close to the temperature *M*_s_. This process indicates the formation of bainite initiated by midrib. The maximum number of medium energy events are generated in different time intervals depending on the transition temperature. The reason for this second recorded process is the formation of lower bainite with midrib. The increase in the generation of low acoustic energy events over a longer period of time indicates the identification of the acoustic background.

Analogous graphs generated during research on the transformation in C105U steel show that high-energy events are generated approximately 8–10 s after the start of the entire process. The probable source of the acoustic signal for temperatures near and below the *M*_s_ temperature is the formation of midrib, which is a twinned thin-plate martensite. The highest intensity of the generation of medium energy events in the tested process for the signal obtained during austempering of steel at a temperature of 100 °C is in the range of 20–30 s, and at a temperature of 130 °C, in the range of 30–35 s. In the following time intervals, the intensity events are rapidly decreasing.

### 3.2. Results of Microstructural Tests

SEM images were taken from areas considered representative of the entire samples. Imaging locations were selected after prior observation of the microstructure under an optical microscope, which allowed exclusion of regions with local defects or mechanical damage. All samples were prepared using the same metallographic procedure, which included cutting, progressive grinding (up to 2000 grit), final polishing with diamond pastes (1 µm), and thorough cleaning in isopropyl alcohol. The etching process was carried out using the same reagent and identical timing for each sample to ensure full repeatability. The sample preparation procedure had been previously optimised to minimise the risk of artifacts such as over-etching, discolouration, or other microstructural distortions. This approach successfully minimised variability and potential disruptions in the interpretation of results.

**Figure 7:**
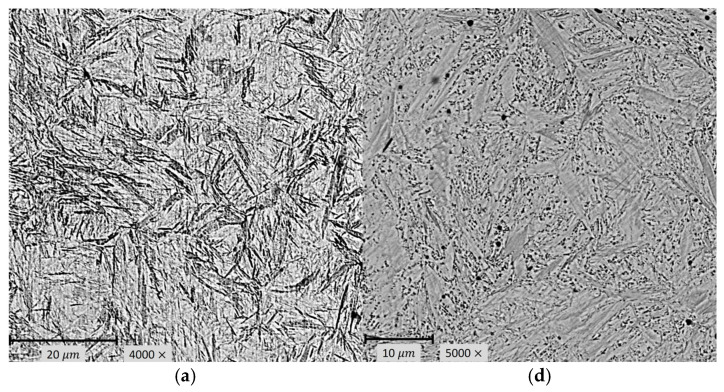

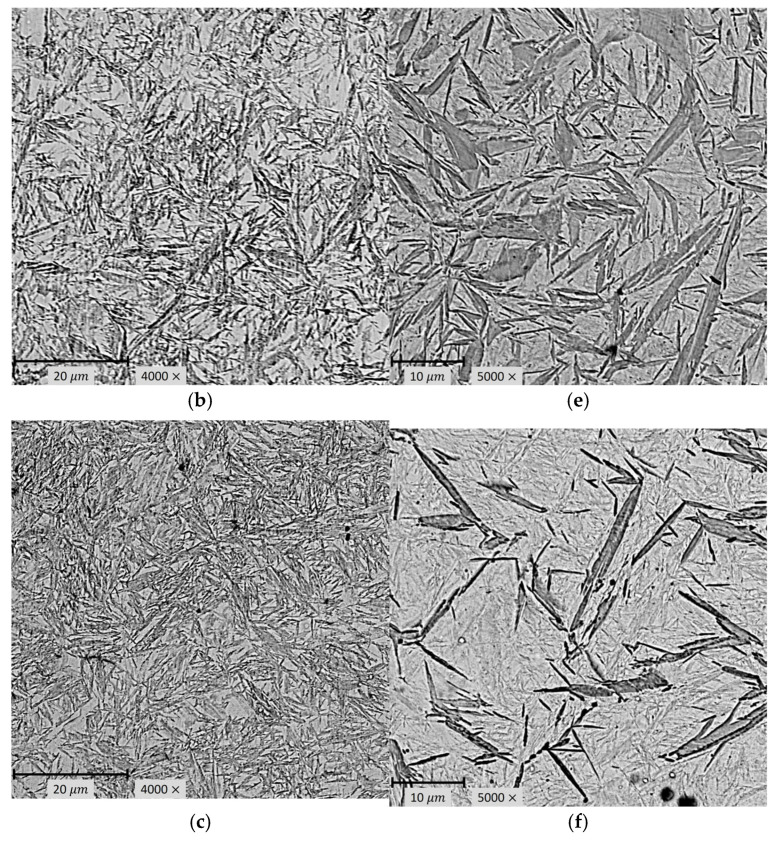
Images of the microstructure after austempering of alloy steel at 130 °C (**a**), 160 °C (**b**), and 180 °C (**c**), and non-alloy steel at 100 °C (**d**), 130 °C (**e**), and 160 °C (**f**) after etching the matrix with Vilella reagent, using a scanning microscope and metallographic microscope. Visible etched: lower bainite with midrib, isothermal martensite, and retained austenite.

Microstructural analysis of alloy and non-alloy steel subjected to austempering in the temperature range of 100–180 °C was carried out using a Phenom XL scanning electron microscope (Thermo Fisher Scientific, Waltham, MA, USA). Microscopic examination enables the comparison of images of the microstructure after austempering with the isothermal transformation after etching of the bainitic and martensitic structure with Vilella reagent, analysing the surface condition, and assessing its morphology. The results of microstructural tests are presented in Figure 7a–f.

## 4. Conclusions

Problems with identifying phase transformations occurring during steel hardening result from the complexity of thermodynamic processes occurring in the volume of the hardened object and the formation of various transformation products at the same time. The above-presented test results of the acoustic signal and the proposed method of its processing turned out to be effective in tracking the above-mentioned phenomena. Events with different energies and spectral characteristics were detected in the recorded AE signal. Based on the determined characteristics of AE events in the recorded signal records, characteristic stages of the studied transformation were identified.

The applied heat treatment process was austempering (with a temperature hold at the specified temperatures). The temperatures were selected based on the Ms temperature (martensite start) for the steels used (approximately 160 °C for the alloy steel and approximately 130 °C for the non-alloy steel). Consequently, tests were carried out below the Ms temperature, within the Ms temperature range, and above the Ms temperature. The research particularly focused on monitoring changes in the acoustic emission signal and identifying the start and end of the phase transformation. This is crucial for a complete understanding of the phenomena occurring during phase transformations. Within this temperature range, bainitic and martensitic transformations occur. The bainitic transformation is a diffusional process—it occurs at lower temperatures than the pearlitic transformation but higher than the martensitic transformation. The bainitic transformation, proceeding through a shear mechanism, offers a favourable balance between strength and ductility. The martensitic transformation is a diffusionless process—a rapid and intense shift of the austenite crystal lattice into the tetragonal structure of martensite. This results in increased hardness and strength but also significant brittleness (leading to the production of very hard components).

The use of an artificial neural network to analyse the AE signal made it possible to separate the ongoing processes. Based on the determined spectral characteristics, feature patterns were generated and used in the network training procedure. The neural network prepared in this way was able to classify the signals fed into its input in terms of their similarity to the learned patterns. Using this procedure, the frequency of occurrence of characteristic AE events was determined as a function of time in order to monitor processes and identify phase changes occurring in the tested material.

The acoustic emission method using artificial neural networks can be successfully used to study phase transformations occurring in steel. Monitoring heat treatment processes has a significant impact on the utility value of finished industrial products. By tracking the kinetics of the decomposition of subcooled austenite, industrial processes can be regulated and improved. Knowing the start and end of phase transformations can improve production efficiency (by shortening heat treatment time) and reduce costs. Optimising processes can enable economical use of time without compromising the quality of manufactured products. The use of methods enabling control of the beginning and end of phase transformations may therefore contribute to the optimisation of industrial processes in the future.

At temperatures of 130 °C for tool steel and 160 °C for bearing steel, the maximum acoustic activity of the process was observed. In this temperature range, close to *M*_s_ for both steels accepted for testing, there is a dominant area of transformation of subcooled austenite to lower bainite with midrib.

The phase transformation occurs in two stages. The first stage of the transformation is the nucleation of thin-plate martensite in the form of midribs. In the second stage, bainite nucleates on the midribs, enriching the austenite with carbon and, consequently, creating lower bainite. Midrib is a twinned thin-plate martensite, and it is the first element of the bainite plate forming in terms of the accelerated onset of transformation. The “butterfly” morphology is characteristic of lower bainite with midrib, which is observed in the microstructural images of C105U steel.

The duration of the bainitic transformation in the tested temperature range depends strictly on the austempering temperature of the steel. As the temperature increases to *MS* of the tested steels, a prolonged transformation duration is observed. The dominant spectrum range of the AE signal generated during the tests is between 100 and 300 kHz. Moreover, the maximum spectral density is observed at a frequency of 180–200 kHz. The spectrograms show that, regardless of the steel grade, a significant number of high-energy AE events are recorded at the beginning of the hardening process.

Software identifying AE events with different energy values made it possible to separate AE events originating from phase transformations, and also to track the kinetics of transformations occurring during steel hardening.

An extensive population of events with various energy values was identified in the examined AE signal. In order to effectively analyse this signal, it was necessary to determine patterns of spectral characteristics that were then used to identify the stages of the ongoing phase transformation.

The method based on acoustic emission (AE) and neural networks demonstrated effectiveness under laboratory conditions. However, it is important to note that there are certain limitations (in the form of potential sources of error) when applying this method, particularly in industrial environments. AE systems require precise placement of acoustic sensors. Applying this method to large components or complex structures may present additional challenges in data acquisition and analysis. Furthermore, there is a risk of overfitting the network, especially when the number of training samples is limited. This can result in overly optimistic outcomes during internal testing but weaker performance in real-world applications. Parameters such as the material’s chemical composition, sample size, and heating/cooling rates affect the characteristics of the AE signal. In industrial settings, mechanical, electrical, and acoustic noise can interfere with the proper detection of signals specifically related to phase transformations. This necessitates more advanced signal filtering or additional verification methods. The phase transformation analysis method described in this work shows promise, but further research is needed to improve its robustness against disturbances and enhance its versatility under variable industrial conditions.

## Figures and Tables

**Table 1:** Chemical composition of 100CrMnSi6-4 bearing steel, element content in wt%.

C	Mn	Si	P	S	Cr	Ni	Mo	Al	Cu
0.9	1.2	0.7	0.01	0.01	1.6	0.08	0.02	0.02	0.20

**Table 2:** Chemical composition of C105U tool steel, element content in wt%.

C	Mn	Si	P	S	Cr	Ni	Mo	V	Cu
1.1	0.3	0.25	0.02	0.02	0.15	0.07	0.01	0.002	0.08

## Data Availability

The original contributions presented in this study are included in the article. Further inquiries can be directed to the corresponding author.

## References

[1] Šmak R., Votava J., Lozrt J., Kumbár V., Binar T., Polcar A. (2023). Analysis of the Degradation of Pearlitic Steel Mechanical Properties Depending on the Stability of the Structural Phases. Materials.

[2] Esmaeilzadeh R., Pandiyan V., Van Petegem S., Van der Meer M., Nasab M.H., de Formanoir C., Jhabvala J., Navarre C., Schlenger L., Richter R. (2024). Acoustic emission signature of martensitic transformation in laser powder bed fusion of Ti6Al4V-Fe, supported by operando X-ray diffraction. Addit. Manuf..

[3] Li Y., Xiao G.-Y., Chen L.-B., Lu Y.-P. (2014). Acoustic emission study of the plastic deformation of quenched and partitioned 35CrMnSiA steel. J. Miner. Metall. Mater..

[4] Schabowicz K., Gorzelańczyk T., Szymków M. (2019). Identification of the Degree of Degradation of Fibre-Cement Boards Exposed to Fire by Means of the Acoustic Emission Method and Artificial Neural Networks. Materials.

[5] Trafarski A., Łazarska M., Ranachowski Z. (2021). Application of acoustic emission to the analysis of phase transformations in 27MnCrB5-2 steel tests during continuous cooling. Bull. Pol. Acad. Sci. Tech. Sci..

[6] Łazarska M., Musiał J., Tański T., Ranachowski Z. (2024). Transformations in the Ti-6Al-4V alloy studied using dilatometry supported by acoustic emission. Materials.

[7] Van Bohemen S.M.C., Sietsma J., Hermans M.J.M., Richardson I.M. (2007). Analysis of acoustic emission signals originating from bainite and martensite formation. Philos. Mag..

[8] Voronenko B.I. (1982). Acoustic emission during phase transformations in alloys. Met. Sci. Heat Treat..

[9] Planes A., Mañosa L., Vives E. (2013). Acoustic emission in martensitic transformations. J. Alloys Compd..

[10] Pieczyska E.A., Tobushi H., Takeda K., Stróż D., Ranachowski Z., Kulasiński K., Kúdela S., Luckner J. (2012). Martensite transformation bands studied in TiNi shape memory alloy by infrared and acoustic emission techniques. Kov. Mater. Met. Mater..

[11] Pawełek A., Kuśnierz J., Bogucka J., Jasieński Z., Ranachowski Z., Ranachowski P., Rajmund F., Dębowski T. (2007). Acoustic emission and the Portevin—Le Châtelier effect in tensile tested Al alloys processed by ARB technique. Arch. Acoust..

[12] Davenport E.S., Bain E.C. (1930). Transformation of Austenite at Subcritical Temperatures. Metall. Class..

[13] Okamoto H., Oka M. (1986). Lower Bainite with Midrib in Hypereutectoid Steels. Metall. Mater. Trans. A.

[14] Oka M., Okamoto H. (1988). Swing Back in Kinetics Near Ms in Hypereutectoid Steels. Metall. Trans. A.

[15] Bhadeshia H.K.D.H., Christian J.W. (1990). Bainite in Steels. Metall. Trans. A.

[16] Yang Z.-G., Fang H.-S. (2005). An overview on bainite formation in steels. Curr. Opin. Solid State Mater. Sci..

[17] Bhadeshia H.K.D.H. (2001). Bainite in Steels.

[18] Haykin S. (1994). Neural Networks: A Comprehensive Foundation.

